# Supplementary material for: Comparison of conventional versus customised Eurosil-4 Pink bolus for radiotherapy of the chest wall
Source: PLoS One. 2022 May 5;17(5):e0267741. doi: 10.1371/journal.pone.0267741 (PMC9071134; doi:10.1371/journal.pone.0267741)
Supplement: S1 File — (PDF) [file pone.0267741.s001.pdf]

Supporting Information

S1: Materials

S1.1 3D-Printers

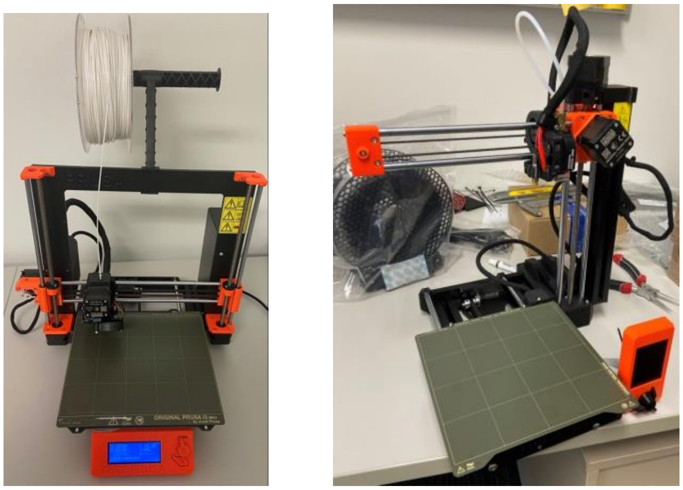

Figure S1: Prusa i3 MK3S (left) Prusa MINI (right)

The project implemented the use of Prusa i3 MK3S 3D-Printer and Prusa MINI 3D-Printer (Prusa Research, Czech Republic). Both of these 3D-Printer models have the feature of automatic calibration for XYZ axes, first layer, temperature, mesh bed leveling, etc. The filament can also be loaded automatically. Firmware upgrades can be used to add new features any time after the purchase of the printer. The Prusa i3 MK3S 3D-Printer has a larger build volume (25 × 21 × 21 cm) in comparison to Prusa MINI 3D-Printer (18 × 18 × 18 cm). It can support all thermoplastic filaments (including Nylon and Polycarbonate), whereas Prusa MINI 3D-Printer can only support PLA, PETG, ASA, ABS and Flex. For this research, PLA and PETG were used for printing.

S1.2 3-D Printing Software

PrusaSlicer (Prusa Research, Czech Republic): https://help.prusa3d.com/en/article/download-prusaslicer_2220/

PrusaSlicer is a slicing software compatible with Prusa 3D-Printers. It is used to convert the 3D models from STL (stereolithography) file format into G-code. Its major benefits are custom print settings, filament settings and printer settings.

3DSlicer (Brigham and Women's Hospital, Boston, MA, USA): https://download.slicer.org/

3D Slicer offers efficient analysis of medical images. The software contains over 100 built-in modules and more can be installed from extension manager. DICOM files can be imported from the DICOM module. The data can be viewed in 3D from anterior, posterior, superior as well as inferior orientations. Volume rendering module helps to display the 3D volume of the data. The segment editor module allows the user to perform contouring of anatomical structures. The segmented data can be converted to 3D surface models using the model maker module. Other important modules are markups, registration, quantification, diffusion, etc.

Meshmixer (Autodesk, San Rafael, CA, USA): https://www.meshmixer.com/

Meshmixer helps in cleaning up and modifying 3D meshes from an STL file.

Fusion 360 (Autodesk, San Rafael, CA, USA): https://www.autodesk.com/products/fusion-360/free-trial

Fusion 360 is useful in designing and engineering 3D models. It's a computer aided design (CAD) software which enables the user to build structures with accurately known dimensions and geometries.

S1.3 Eurosil-4 Pink

Eurosil-4 Pink (SynTec, Schouten Group, Netherlands) is used as a silicone mould making tool. It has two components (A and B) that need to be added together. It should not take more than 10 minutes so as to avoid the substances from solidifying before the required quantity is poured. Both the components are used in the ratio of 1:1. The mixture is then stirred till it looks uniform and left out for 24 hours after that. The mould material has high tensile strength (4.5-5 N/mm^2) and tissue equivalence (density is 1.04 g/cm^3). It also has good skin compatibility.

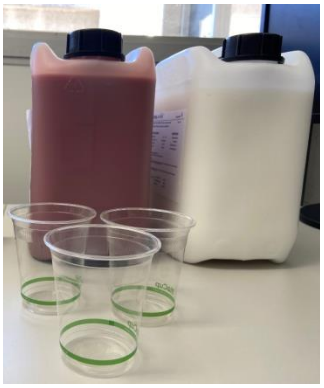

Figure S2: Component A (white) and component B (pink) of Eursoil-4 Pink

S2: Methods

S2.1 Measured thickness and density for flat Eurosil-4 Pink boluses

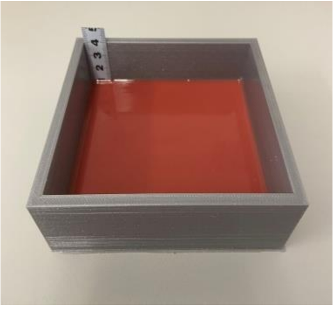

Figure S3: Filling the tray with Eurosil-4 Pink to construct bolus

Autodesk Fusion 360 was used to model a tray with an internal volume of 10 cm × 10 cm × 4 cm. A scale was glued at one corner to measure height. The two components of Eurosil-4 Pink were mixed well and poured in the container till the height of 1 cm. This was left to dry for 24 hours. When extracted out, a bolus of base 10 cm × 10 cm and height 1 cm was obtained. The same procedure was followed to make boluses with the same base, and heights of 0.5 cm, 1 cm, 2 cm, and 3 cm. The bolus thicknesses were measured using a vernier caliper, 10 times for each of the 4 boluses. The thicknesses calculated were 0.56 ± 0.12 cm, 0.93 ± 0.11 cm, 2.09 ± 0.21 cm and 2.81 ± 0.17 cm. For the bolus thickness experiment, the boluses can be stacked together to get a different thickness.

A CT scan of all four boluses was taken to check if they have a uniform density. The DICOM files were imported in 3D Slicer. After volume rendering, the threshold was set as the range 47.28 to 2061.95 HU. Then using the scissors tool in the same module (with settings: operation - erase

outside; shape - rectangle; slice cut - unlimited) a cuboidal segment was selected from the bolus, excluding the edges and corners. The edges were not included so as to avoid beam hardening CT artefact. The segment statistic module was used to calculate the mean and standard deviation of Hounsfield units (HUs) of the segment for each bolus. We got 249.73 ± 19.90 HU for 0.5 cm bolus, 239.30 ± 12.41 HU for 1 cm bolus, 244.24 ± 16.67 HU for 2 cm bolus and 241.30 ± 14.22 HU for 3 cm bolus. This confirmed that the boluses had a uniform density.

S2.2 Eurosil-4 Pink sheet to insert ion chamber

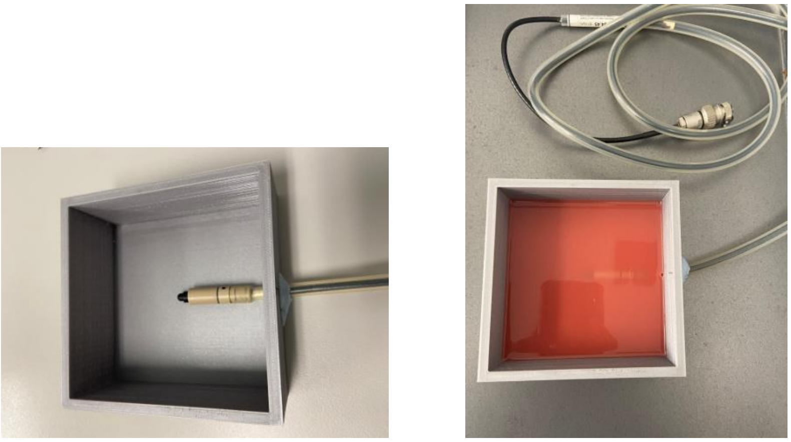

Figure S4: Process for construction of ion chamber sheet

The CC04 ionisation chamber was added in the setup with the help of one Eurosil-4 Pink sheet that had an insert for the ion chamber. The Eurosil-4 Pink sheet was preferred for inserting the ion chamber instead of solid water because it is the same material as the Eurosil-4 Pink boluses; thus more suitable for dose calculations. In order to construct this sheet, a hole of the size of ion chamber was drilled in the tray which was previously used for bolus construction. The ion chamber was fitted such that it would be close to the surface of the sheet. Eurosil-4 Pink was filled in and left to dry for 24 hours.

S2.3 Setup for dose delivery (Air gap analysis)

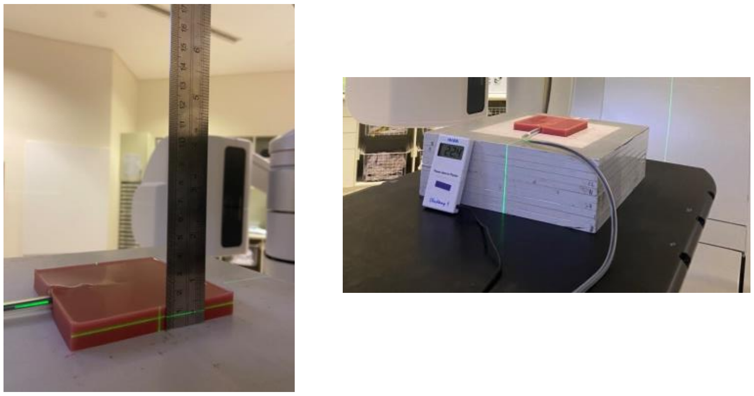

Figure S5: Setting up the SAD and other setup parameters

10 cm of solid water was kept on the LINAC couch for backscatter, and the Eurosil-4 Pink sheet containing the ionisation chamber was centered on it with the light field. The temperature was measured with a thermometer. The SAD was set as the distance from the source to the center of the height of the ion chamber sheet, and was equal to 100 cm. A 6 MV beam was used in all the measurements.

S2.4 Checking optimal bolus thickness

At first, the setup was irradiated without any added bolus. Afterwards, irradiation was performed for separate cases of the following thickness of added bolus: (i) 0.5 cm (ii) 1 cm (iii) 1.5 cm (iv) 2 cm (v) 2.5 cm (vi) 3 cm (vii) 3.5 cm (viii) 4 cm. 200 MUs were delivered and the field size was 10 cm × 10 cm. Three readings were taken from the electrometer for each case.

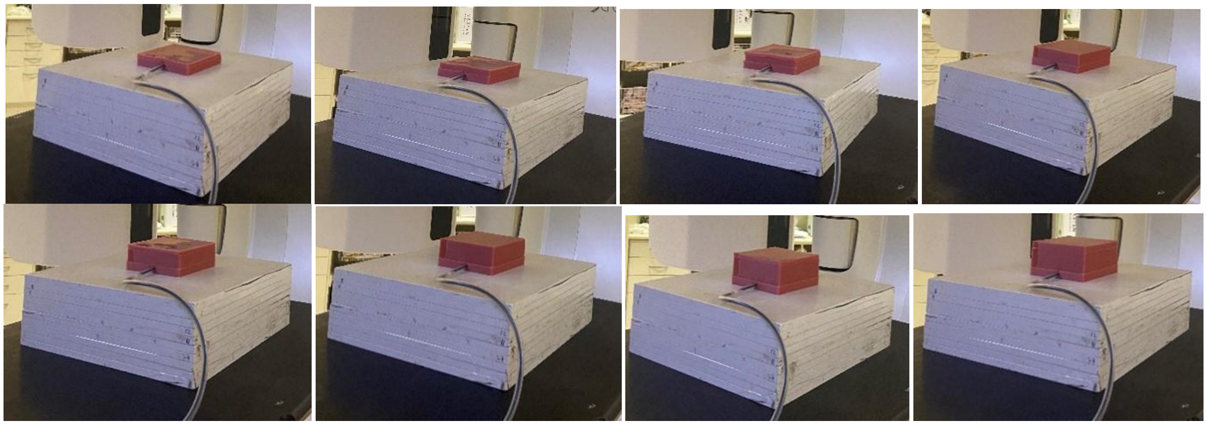

Figure S6: Boluses of different thickness irradiated separately

S2.5 Air gap analysis

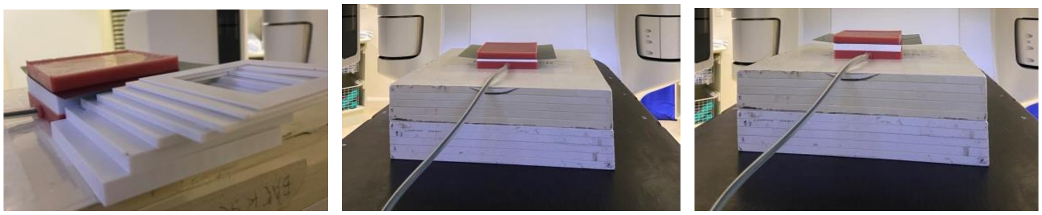

Figure S7: Varying thickness of air gaps inserted

The air gap was inserted between the ion chamber sheet and the bolus. A used radiochromic film was inserted on top of the air gap stand to prevent the bolus from bulging into the air gap, and the films thickness was considered in the calculations. Irradiations were performed separately for no air gap used and the different cases of air gaps (i) 0.1 cm (ii) 0.2 cm (iii) 0.3 cm (iv) 0.4 cm (v) 0.5 cm (vi) 0.8 cm (vii) 1 cm (viii) 1.5 cm. 200 MUs were delivered, and field size was 10 cm × 10 cm. Three readings were recorded from the electrometer for each case.

In the next part, the field size was set to the average field size found for the patient plan, i.e., 2.8 cm × 22.7 cm. The same setup was repeated, and dose was delivered for each air gap separately. 50 MUs were used, and three readings were recorded for each case.

In order to observe the effect of oblique beams, measurements were performed for the following angles of incidence- 0°, 10°, 20°, 30°, 40°, 50°, and 60°. The field size was same as the last part of the experiment, i.e., 2.8 cm × 22.7 cm and the gantry angle was changed to a given

angle. This was done for the case of no air gap and 1 cm air gap. Three readings were taken for each angle of incidence.

S2.6 Construction of customized Eurosil-4 Pink bolus

The patient CT was imported into 3D Slicer software. First, volume rendering module was used to visualize the 3D volume of the patient. A label map was created in the editor module by using the threshold effect tool, which selects the region to be included in the model. The threshold should encompass the entire chest surface. The make model effect tool was used to convert the chest label map into a 3D model, which was then saved in STL file format.

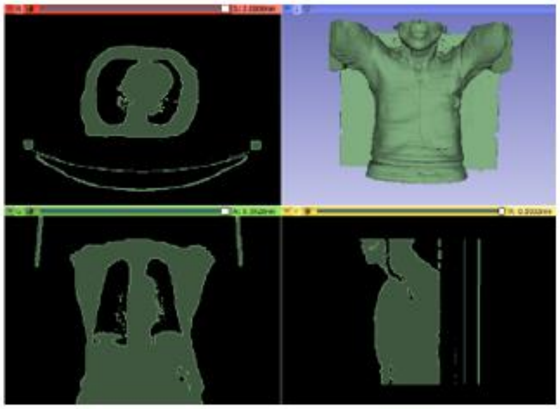

Figure S8: Model making in 3D-Slicer after volume rendering

The chest 3D model was imported into Meshmixer software. A surface region covering the tumour was selected, which was located on the chest wall. This was then extracted from the entire chest as a separate 3D layer. As this is the only layer which is required for making the bolus, the rest of the model can be cleared from the window. The 3D layer was extruded in the normal direction by a distance equal to the desired thickness of the customised Eurosil-4 Pink bolus. It was then cut to the required shape, and smoothed to remove the artefacts and CT slice irregularities. The modified file was saved separately as ’bolus shell.stl’.

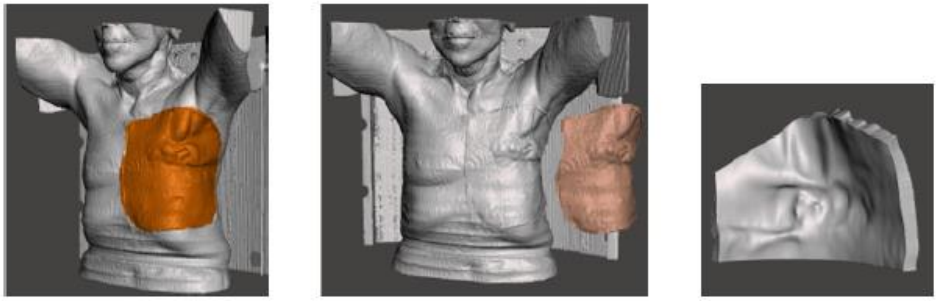

Figure S9: Extracting a surface layer in Meshmixer

’bolus shell.stl’ was imported in Prusa Slicer (Prusa Research, Czech Republic). The print settings were set as 0% infill, spiral vase, 1 raft layer, and PLA was chosen as the filament. A 3D-printed hollow bolus shell was obtained, which was then filled with Eurosil-4 Pink. After 24 hours the shell was cracked open to get the Eurosil-4 Pink bolus, customized to the patient chest surface. Two thicknesses of Eurosil-4 Pink bolus (0.6 cm and 1.1 cm) were made from this procedure.

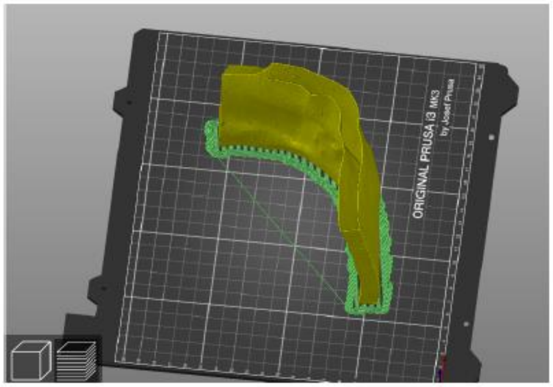

Figure S10: Adjusting print settings in PrusaSlicer (Such as 0% infill)
